# Sociopetal design in psychiatric therapuetic settings

**DOI:** 10.1192/j.eurpsy.2023.172

**Published:** 2023-07-19

**Authors:** J. Danziger

**Affiliations:** Design, thinkbuild architecture BDA, Berlin, Germany

## Abstract

**Abstract:**

Sociopetal design methods can offer interesting means to support therapeutic concepts within ward environments. They can help to forge group identities through offering patients, staff and visitors opportunities to identify with the spaces they inhabit. “Sociopetal space” has been defined as “spaces which help bring people together"; but how does this actually work and what role can these types of spaces play in a hospital ward setting?

Some of these elements operate at a detail level and can be rather simple to deploy. Normalising the environment by making “regular” design decisions such as by using real rather then simulated materials (ie., actual wood rather then “wood patterned” furniture); or through offering a mix of lighting (ie., artificial and natural sources in variation) can create more homely spaces for patients and staff alike. Ultimately, design decisions at the detail scale can create phenomenal elements which can play a large role towards generating a favorable atmospheric experience on the ward.

It is also possible to explore how specific moments or places within a psychiatric ward might be designed to support patient agency, even on a closed ward. Sociopetal elements such as well-sited sitting spaces can offer moments of safety or retreat, leading to a greater sense of control. This can help patients feel more open to positive interactions with their colleagues and staff because they can safely observe or choose less committed ways of participation in daily or group activities.

Zooming out from these details, we will also look at the layout of a psychiatric ward (ie. accommodations) to help identify where opportunities such as those listed can be found. Simple gestures such as a slight widening of the corridor leading to important shared areas or better access to light or views of nature have been shown to improve outcomes for patients. What other design elements can be placed on or within wards to further this approach? Recent and ongoing projects within our practice will be shared to help workshop participants gather literacy in case they may be involved in future design projects.

**Disclosure of Interest:**

None Declared

